# Low Cost Continuous Femoral Nerve Block for Relief of Acute Severe Cancer Related Pain Due to Pathological Fracture Femur

**DOI:** 10.4103/0973-1075.73669

**Published:** 2010

**Authors:** Rachel Cherian Koshy, G Padmakumar, O Rajasree

**Affiliations:** Department of Anaesthesiology, Regional Cancer Centre, Thiruvananthapuram - 11, Kerala, India

**Keywords:** Cancer pain, Continuous femoral nerve block, Pathological fracture femur

## Abstract

Pathological fractures in cancer patient cause severe pain that is difficult to control pharmacologically. Even with good pain relief at rest, breakthrough and incident pain can be unmanageable. Continuous regional nerve blocks have a definite role in controlling such intractable pain. We describe two such cases where severe pain was adequately relieved in the acute phase. Continuous femoral nerve block was used as an efficient, cheap and safe method of pain relief for two of our patients with pathological fracture femur. This method was proved to be quite efficient in decreasing the fracture-related pain and improving the level of well being.

## CASE 1

A fourteen-year-old girl underwent open biopsy for suspected osteosarcoma femur. An intraoperative C-arm examination revealed a pathological fracture at the lower end of femur. Balanced general endotracheal anesthesia was administered for the biopsy procedure. She was premedicated with oral alprazolam 0.25 mg at bedtime the previous day and on the morning of surgery and induced with Inj Midazolam 1mg, 1% Inj Propofol 100 mg, 100 microgram Inj Fentanyl and 25 mg Inj Atracurium and maintained with Nitrous Oxide, Oxygen and Isoflurane. The operative procedure lasted for 45 min. During the intraoperative period, she was also given intramuscular Diclofenac 50mg. Following the procedure, muscle relaxation was reversed and trachea was extubated.

The patient was in severe pain on arousal in spite of the adequate opioids and NSAID. Post operatively the patient with a VAS 10/10 was started on infusion Fentanyl with a PCA pump. Her pain did decrease but she lacked the confidence to sit up, eat, pass urine and so on. She was terrified of the movement-related pain.

The following day a femoral block was performed on the patient with the nerve being identified by the patellar dance using a nerve stimulator. The sheath of an ordinary intravenous cannula was left *in situ* at the identified femoral nerve site. This was connected to a 10 cm extension line and anchored in place with support of small sterile gauze and sutures. Ten milliliter of 1% Lignocaine was given. The patient had immediate pain relief with VAS of 0/10 reflected by a smile on her face. This also helped her to regain the lost confidence. Following the procedure, she felt comfortable to move, sit up and use the bed pan. Such quality of pain relief was not obtained with escalating doses of potent analgesics without side effects. She became more compliant and was willing to co-operate for further treatment. The bolus was followed with continuous infusion at 3ml per hour using 0.25% Bupivacaine.

## CASE 2

A 42-year-old male was operated for carcinoma parathyroid. He had brittle bones. Despite extreme care during shifting to and from surgery, he developed pathological fracture of femur which produced severe pain in the post operative period. He had a pain score of 10/10 (VAS) which did not subside adequately with intravenous analgesics (Morphine, Diclofenac, Tramadol in maximal allowable doses).

Hence a femoral nerve block was done with an insulated needle passed through the sheath of an ordinary 18G intravenous cannula. Peripheral nerve stimulator was used to elicit movement of the patella. A bolus dose of 25 ml of local anesthetic (0.25% Bupivacaine) provided immediate complete pain relief and provided a hope of cure and the ability to walk normally in the course of time. The bolus injection was repeated every 6 h providing adequate and complete pain relief.

To alleviate the patient’s anxiety, we used midazolam 20μg/kg given as an intravenous bolus 1 min before the placement of the needle in both cases.

### Method of Block

The femoral nerve is situated lateral to the femoral artery and is deep to the iliaca fascia, which in turn is deep to the fascia lata. The femoral artery and vein are in a separate fascial compartment. The femoral nerve is one nerve bundle near the inguinal crease, but a short distance more distally it divides into its two branches (superficial and deep branches).

The femoral artery is marked in the groin with a permanent marker. The femoral nerve is situated 1–2 cm lateral to the femoral artery. Mild sedation is all that is required for this block.

The equipments required include an 18 gauge PVC cannula, insulated needle which deliver current at the tip with a port to deliver the drug (Stimuplex needle), one ECG electrode, peripheral nerve locator or stimulator (with internal mode).

The patient lies supine with a clear view of the patella. The groin is prepared with Betadine and draped with a sterile drape.

After careful skin and subcutaneous tissue infiltration of local anesthetic agent (care must be taken not to block the femoral nerve in the process), the cannula sheath is thread over the stimulating insulated needle and inserted aiming approximately 45 degrees cephalad just inferior to the inguinal crease. The nerve stimulator is connected to wire at the proximal end of the needle.[1] After the nerve has been located with the cannula threaded over the needle till the dancing movement of the patella disappears signifying the loss of contact of the bare needle with the nerve due to the cannula. The cannula is withdrawn few millimetres till we can once again elicitate the muscle twitches and patellar movement unchanged in character and intensity. The catheter is now correctly placed near the femoral nerve but will most likely dislodge over time unless secured. At that point, the cannula is fixed to the skin with suture material and proper adhesive plaster.

The nerve stimulator is once again stimulated to confirm the position of cannula and the needle. Optimal positioning evoke contractions with 0.5mA or less and the evoked response fades after administration of 1-2ml of local anesthetic drug (Raj test).[2] The needle is then removed from the cannula and an extension line of 10 cm attached to the proximal end.

Initial bolus of 10 ml local anesthetic (1-2% xylocaine) is to be administered.

Breakthrough pain is rare and patient satisfaction is high in patients when an infusion of 0.1ml/kg/h in children or 5ml/h in adults of 0.25% bupivacaine is used.

The entry site of the catheter should be inspected daily for any signs of infection. Since an indwelling cannula is left *in situ* formal sterile procedures were advised as other indwelling catheter. Sensation is allowed to return to the limb before the catheter is removed and if the patient complained of pain a bolus dose of the drug was administered or the infusion was maintained for few more days. In our cases, the cannula was removed on the fourth day of the catheter placement with no signs of infection.[3]

We had seen that this method of cannula placement was quite effective with minimal adverse effects and above all cost effective.

The cannula should always be withdrawn entirely into the needle before the needle is repositioned. The presence of significant paresthesia during cannula advancement should be carefully evaluated before advancement of the cannula^2^. Be suspicious of sub-perineural needle or cannula placement if brisk muscle twitches are present with nerve stimulator settings less than 0.2mA (except in children).[2] Since an indwelling cannula is left *in situ* for some time, formal sterile procedures are necessary. Sensation should be allowed to return to the limb before the cannula is removed.

## DISCUSSION

Continuous femoral analgesia provides extended pain relief and improved functional recovery for patients with fracture femur.[4–6] Postoperative analgesia was excellent with a median VAS score of 1 (0–7) at the fourth hour and that did not increase throughout the block. It was seen that the extent of pain relief was comparable to that obtained with continuous femoral nerve block by use of stimulating catheters which was more costly than a simple intravenous cannula.[6] Successful continuous peripheral nerve analgesia depends on the catheter proximity to the target nerve. If the catheter is not close to the nerve, high infusion rates may be required to provide analgesia or analgesia may be sub-optimal. The amount of local anesthetic required for successful femoral nerve blocks was similar in patients receiving continuous femoral nerve block. We have seen that continuous femoral nerve block provides better pain control than IV patient controlled analgesia with morphine or other opioids.[6,7]

Pain during movement caused by electrical stimulations was the main cause of discomfort during the anesthetic procedures. A previous experience with a regional technique is a significant factor in acceptance of this regional technique for future surgery.

Severe complications were rare when catheters are used for continuous nerve blockade. Some studies have reported of major vascular injury and paresthesia when the catheters were left *in situ* for prolonged period.[3]

Potential limitations of femoral nerve blocks include block failure and toxicity from systemic absorption of local anesthetic. The use of a peripheral nerve stimulators helps in the proper location of the nerve and catheter placement. The use of smaller amounts of local anesthetics in continuous femoral nerve blocks is desirable because it reduces potential toxicity without hampering the degree of pain relief.[1,6]

Though stimulating catheters may provide better catheter placement,[1] it appears the above cannula technique is at par in terms of pain relief. Low cost and ease of placement favour the cannula technique for resource poor countries. Commercially available catheters for continuous nerve blocks are expensive. Since the femoral nerve is relatively superficial, it is possible to substitute a cheap intravenous cannula for the expensive commercially available catheter. Attaching a 10cm extension line to the cannula sheath prevents it from being dislodged by handling during top up dosing procedure. The Stimuplex needle also is thoroughly cleaned and ETO sterilised or placed in formaldehyde for reusing repeatedly. We have not come across any infective complication and are able to provide low cost pain relief continuously even for the poorest patient.[3]

We conclude that continuous femoral nerve block for post operative analgesia and fracture femur is effective, with nil complication and high success rate. This continuously relieves the muscle spasm from fracture femur.[8–11]
